# Septic thrombophlebitis of portal and splenic vein secondary to Fusobacterium nucleatum: A case report of an abdominal variant of lemierre syndrome

**DOI:** 10.1097/MD.0000000000035622

**Published:** 2023-10-13

**Authors:** Sagar Pandey, Aditya Keerthi Rayapureddy, Kapilkumar Manvar, Sushma Edara, Gouthami Boddu, Ajit Thakur, Vijay Jaswani

**Affiliations:** a Department of Internal Medicine, One Brooklyn Health- Interfaith Medical Center, NY; b Department of Hematology and Medical Oncology, One Brooklyn Health- Brookdale University Hospital Medical Center, NY; c Department of Internal Medicine, B.P. Koirala Institute of Health Sciences, Dharan, Nepal; d Department of Radiology, One Brooklyn Health- Interfaith Medical Center, NY.

**Keywords:** Fusobacterium nucleatum, lemierre syndrome, sepsis, thrombophlebitis

## Abstract

**Rationale:**

Septic thrombophlebitis of the internal jugular vein also known as Lemierre syndrome occurs secondary to an oropharyngeal infection often leading to septic embolisms to distant sites. Anaerobic gram-negative bacillus, Fusobacterium nucleatum and Fusobacterium necrophorum are commonly isolated organisms. Fusobacterium species has also been reported to complicate an intra-abdominal infection leading to septic thrombophlebitis of portal vein also known as pylephlebitis or abdominal variant of lemierre syndrome.

**Patient concerns:**

The patient was a middle-aged female patient with chief complaints of abdominal discomfort, intermittent fever and vomiting for one month.

**Diagnoses:**

The final diagnosis was septic thrombophlebitis of portal and splenic vein secondary to Fusobacterium nucleatum.

**Interventions:**

Patient was managed with broad spectrum intravenous antibiotics with coverage against gram-negative bacilli, anaerobes, and aerobic streptococcus species with therapeutic anticoagulation.

**Outcomes:**

Patient gradually improved and was discharged on oral apixaban. She was instructed to follow up with gastrointestinal specialist upon discharge in anticipation of the need for liver transplant in future.

**Lessons:**

Due to its high mortality and associated long term disease morbidity, clinicians should always strive towards early diagnosis and treatment of the condition with involvement of multidisciplinary teams.

## 1. Introduction

Lemierre syndrome (LS) is one of the rare life-threatening conditions presenting as septic thrombophlebitis of the internal jugular vein secondary to an oropharyngeal infection often leading to septic embolisms to distant sites. Anaerobic gram-negative bacillus Fusobacterium nucleatum and Fusobacterium necrophorum are commonly isolated organisms in patients with LS.^[1]^ Although rare, Fusobacterium has also been reported to complicate an intra-abdominal infection leading to septic thrombophlebitis of portal vein also known as pylephlebitis or abdominal variant of LS.^[2]^ Its incidence rate is reported to be approximately 0.37 to 2.7 cases per 100,000 person years.^[3]^ Endothelial injury, venous stasis and hypercoagulability secondary to inherited or acquired thrombophilic disorders, cirrhosis, myeloproliferative disorders, intra-abdominal inflammatory conditions, abdominal surgery or trauma, malignancy, etc, predispose to the development of portal vein thrombosis.^[4]^ Here, we present a case of infective suppurative pylephlebitis of portal vein and splenic vein with Fusobacterium nucleatum isolated on blood culture in a middle-aged female patient presenting with abdominal discomfort and fever.

## 2. Case presentation

A 46-year-old obese female with a past medical history of type 2 diabetes mellitus presented with complaints of progressive shortness of breath associated with bilateral subcostal discomfort on minimal exertion, intermittent fever with chills and intermittent episodes of vomiting for around 1 month. All the symptoms had an insidious onset. Abdominal discomfort was mild to moderate in intensity, subacute in onset, dull aching, more pronounced on the left subcostal region, non-radiating, with no aggravating or relieving factors. It was associated with occasional episodes of non-bilious, non-bloody vomiting occurring immediately after food intake and containing ingested food particles. Patient also reported intentional weight loss of 150 lbs. over a period of 1 year. Patient denied any history of drug or alcohol abuse along with any history of diarrhea, constipation, or genitourinary complaints. Patient was tachycardic (heart rate of 130), febrile (101.4-degree F) but normotensive (blood pressure of 114/73) at presentation. On examination, the patient appeared toxic and diaphoretic with a soft, non-distended, diffusely tender abdomen especially in the left upper quadrant with no guarding or rigidity. Cardiovascular, respiratory, and neurological examination were within normal limits (except for tachycardia) on examination. Laboratory parameters at presentation are presented in Table 1.

**Table 1:** Laboratory parameters at presentation.

Laboratory parameter	Levels at presentation	Normal reference range
Hemoglobin	10.2	11.4–15.5 gm/DL
Hematocrit	30.3	37–43.7%
White blood cells	15.6	4.5–10.2 10 × 3/UL
Platelets	89	180–401 10 × 3/UL
ALT	38	<35 U/L
AST	75	14–36 U/L
ALP	430	38–126 U/L
Serum albumin	3	3.5–5.0 g/DL
PT	20.1	9.2–12.8 secs
INR	1.78	0.7–1.2
PTT	32.1	23.5–35.5 secs
Serum lipase	63	23–300 U/L
Lactic acid	3.5	0.5–2.2 mmol/L
BUN	32	7–17 mg/DL
Cr	1.5	0.52–1.04 mg/DL

**Figure 1.**
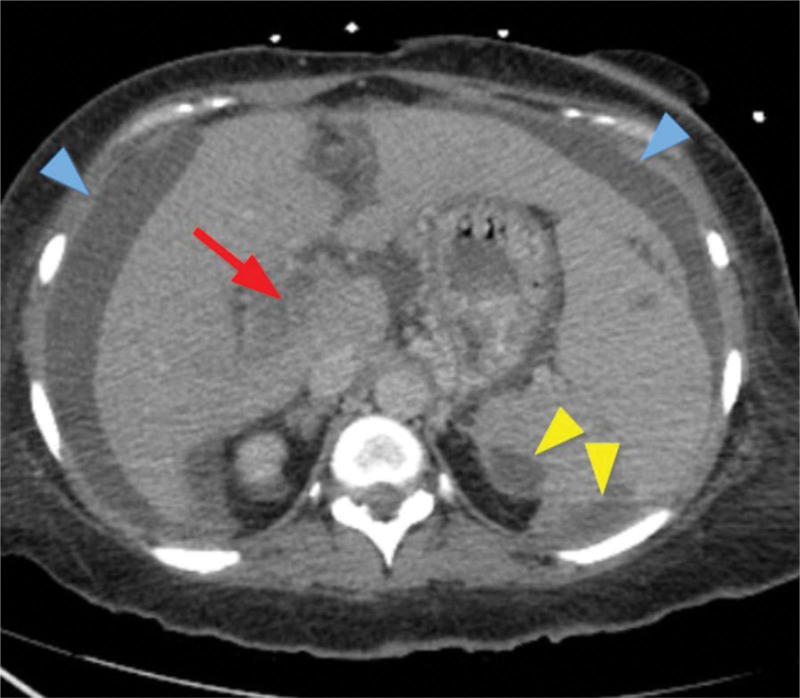
Portal vein thrombus (red arrow), splenic abscesses (yellow arrowheads) with splenomegaly and ascites (blue arrowheads).

**Figure 2.**
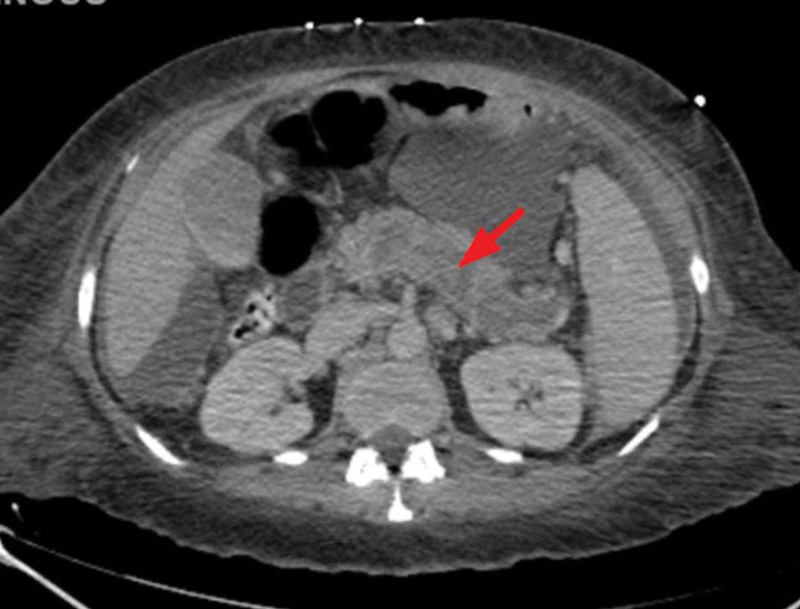
Splenic vein thrombosis.

**Figure 3.**
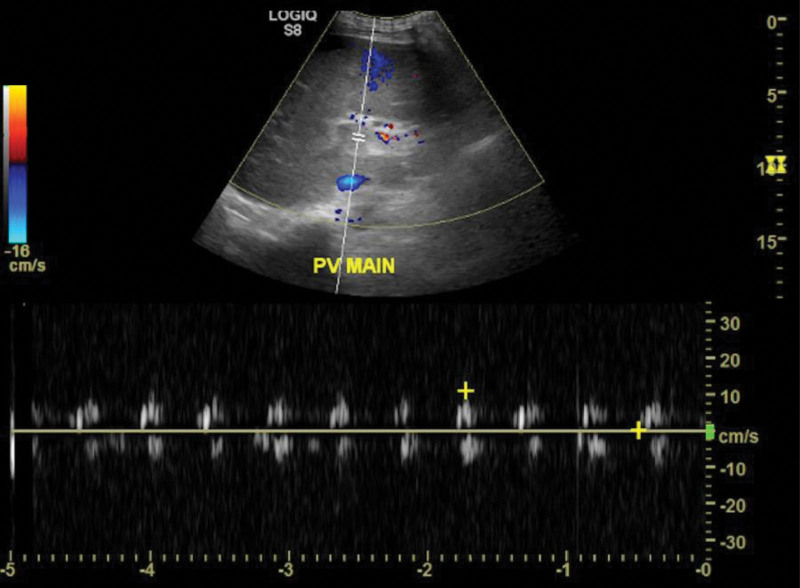
US abdomen with doppler showing absence of flow and abnormal waveform in portal vein indicating a possibility of thrombus.

**Figure 4.**
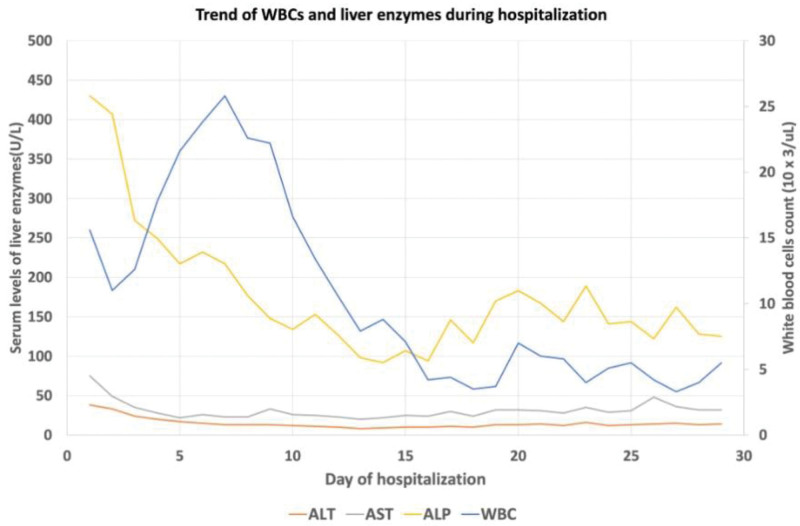
Trend of WBCs and liver enzymes during the length of hospitalization.

Computed tomography (CT) of abdomen and pelvis with intravenous (IV) contrast showed significantly enlarged spleen with subcapsular loculated collection measuring 5 cm × 10 cm concerning for abscess/hematoma (no gas), notable periportal edema with splenic vein tortuosity; short gastric, and splenic hilar varices were also reported concerning for portal hypertension. However, no evidence of hepatic cirrhosis was appreciated (Figs. 1 and 2). Patient was managed with aggressive IV hydration, broad spectrum IV antibiotics for suspected intra-abdominal source of infection (cefepime and metronidazole) and close monitoring in a surgical intensive care unit. Abdominal duplex ultrasonography showed splenic vein thrombosis and portal vein thrombosis (PVT) (Fig. 3). Magnetic resonance imaging abdomen with and without IV contrast further confirmed the presence of acute splenic vein thrombosis/PVT and ruled out gallstone disease, biliary dilation, and pancreatic abnormalities. Patient underwent interventional radiology guided drainage of posterior splenic collection and placement of pigtail catheter which yielded 35 mL of frank pus. With gastroenterology and hematology consultation, the patient was started on therapeutic anticoagulation with enoxaparin and closely monitored for bleeding especially in the presence of gastrointestinal varices. Enoxaparin dose was further adjusted due to extension of thrombus to superior mesenteric vein in subsequent CT scans. Blood culture at presentation grew gram-negative rod, Fusobacterium nucleatum for which the patient was switched to ceftriaxone and metronidazole as per infectious disease recommendations. Fluid from the splenic abscess did not show any growth. IV antibiotics were continued for a period of 6 weeks due to the concern for suppurative thrombophlebitis associated with splenic abscess. A battery of CT imaging showed resolution of abscess following repeated interventional radiology guided drainage of abscesses. Thrombocytopenia seen during hospital stay was attributed likely secondary to splenomegaly and bone marrow suppression secondary to sepsis which subsequently improved with resolution of sepsis. Patient was discharged on oral apixaban 10 mg twice daily for additional 13 doses to complete a total of 6 weeks of therapeutic anticoagulation followed by half the dose (5 mg twice daily) for 3 months. Due to the risk of intestinal ischemia, liver failure and bleeding from varices, patient was instructed to follow up with gastrointestinal specialist upon discharge in anticipation of the need for liver transplant in future. Trend of liver enzymes and white blood cell count during hospitalization is presented in Figure 4.

Infective suppurative thrombosis of portal vein secondary to diverticulitis, appendicitis, cholangitis, infected choledocholithiasis, or acute pancreatitis was ruled out from lack of imaging evidence of respective pathologies. Hypercoagulability workup including protein C, protein S, lupus anticoagulant, anti-cardiolipin, beta-2 microglobulin, homocysteine, factor V Leiden, prothrombin, and antithrombin levels was nonsignificant except for low protein C which was likely transient secondary to progressive thrombosis. A decision to repeat the workup after resolution of acute illness on an outpatient basis was made for an accurate interpretation. Detailed work up for paroxysmal nocturnal hemoglobinuria was not done in the absence of features of hemolysis with normal lactate dehydrogenase, and indirect bilirubin. Myeloproliferative disorders like polycythemia vera, essential thrombocytosis, or primary myelofibrosis were unlikely with negative Janus Kinase 2 mutational analysis. Malignancy was excluded from one of the differentials with lack of imaging evidence in CT chest, abdomen, and pelvis. Furthermore, CA 19–9 and carcinoembryonic antigen were also within normal limits. Liver enzymes, although elevated at presentation, subsequently normalized upon drainage of splenic abscess. Hepatitis screen was negative with a negative history of significant alcohol intake. Furthermore, CT of abdomen showed no evidence of liver cirrhosis. Both indirect immunofluorescence assay and antigen specific enzyme linked immunofluorescence assay for proteinase 3 and myeloperoxidase were negative. Autoimmune vasculitides like granulomatosis with polyangiitis and microscopic polyangiitis were therefore unlikely differentials. Classical LS with septic embolism to the portal system was ruled out due to lack of oropharyngeal symptoms and no imaging evidence of thrombus in the internal jugular vein. Transthoracic echo was negative for any vegetation with no valvular abnormalities except trace tricuspid regurgitation.

## 3. Discussion

Classical LS is diagnosed in the presence of anaerobic primary infection of the oropharynx, septicemia with at least 1 positive blood culture, metastatic septic embolism to distant sites and thrombophlebitis of the internal jugular vein.^[5]^ On the other hand, the diagnosis of pylephlebitis requires the evidence of portal vein thrombosis in a patient with fever and bacteremia. Pylephlebitis commonly presents as an infective suppurative thrombosis of portal vein secondary to intra-abdominal or pelvic infection that is drained by the portal venous system.^[3]^ Portal vein, formed from the confluence of superior mesenteric and splenic vein, drains blood from the spleen, gallbladder, pancreas and gastrointestinal tract (except lower rectum) to the liver.^[6]^ Diverticulitis (26.5%) and appendicitis (22%) are therefore, the 2 most common causes of pylephlebitis followed by pylephlebitis due to unknown cause (11.5%) and that following liver abscess (8.5%), gastroenteritis (6.5%), surgery (6%), pancreatitis (5.5%), inflammatory bowel disease (4%) and others. In a systematic review involving 220 individuals with pylephlebitis, pylephlebitis was secondary to monomicrobial infection in 42.8% of cases, polymicrobial in 27.2% of cases whereas microorganisms were not identified/non-included in the rest of the cases. While Escherichia coli, Bacteroides, and Streptococcus were 3 most commonly isolated bacteria, Fusobacterium species was isolated in mere 10% of cases. Lastly, fever and abdominal pain were the most common symptoms at admission similar to the presentation in this case.^[3]^

Fusobacterium, commonly found as a commensal in oral cavity, gut and female genital tract, possess a myriad of virulence factors responsible for the development of necrotic abscess, septic thrombophlebitis, and thrombosis. For example, bacterial hemolysins lyse the erythrocytes, reducing oxygen transport to the site of infection and creating a favorable anaerobic environment for its growth. Similarly, hemagglutinin activity facilitates platelet aggregation leading to local site thrombosis, heat mediated endotoxin mediates inflammatory response and stimulates production of tumor necrosis factor alpha, lipopolysaccharides in cell wall has strong endotoxic properties, etc.^[5,7]^ A breach in the mucosal surface due to various infectious, inflammatory or neoplastic causes creates a pathway for bacterial invasion which subsequently via direct invasion to local site of infection or lymphatic or hematogenous spread leads to the suppurative thrombophlebitis or distant metastasis of septic embolic.^[5,8]^ Pyelophlebitis begins as thrombophlebitis of small veins draining portal venous system which extends into larger veins leading to portal vein thrombosis; further extension of thrombus can lead to splenic or superior mesenteric vein thrombosis.^[9]^

Isolation of microorganism in blood or body fluid (i.e., aspirate from intra-abdominal abscess) cultures with appropriate clinical history aids in the diagnosis of pylephlebitis. However, pylephlebitis should not be ruled out in culture negative cases especially with evidence of intra-abdominal suppurative process and portal vein thrombosis as negative cultures were reported in 30% of cases of pylephlebitis.^[3]^ CT scan of abdomen and pelvis is often the preferred imaging modality due to its ability to detect venous thrombosis along with intra-abdominal suppurative processes. Empiric intravenous antibiotics for treatment of bacteremia should include coverage against gram-negative bacilli, anaerobes and aerobic streptococcus species which should be subsequently tapered based upon culture and sensitivity results. Given the association of pylephlebitis with intra-abdominal abscess, optimal duration of intravenous antibiotics should be 4 to 6 weeks with or without drainage.^[3,10]^ Due to the lack of large-scale studies on the role of anticoagulation in pylephlebitis, evidence driven recommendations for anticoagulation are lacking. Anticoagulation is generally recommended in patients with progression of thrombosis on repeat imaging, persistent fever despite being on appropriate antibiotics, extension of thrombus beyond the portal vein main branch, or confirmed hypercoagulable state.^[3]^ In a retrospective review of patient records of 67 patients managed for pylephlebitis, it was found that early initiation of anticoagulation yielded long term benefit via prevention of chronic PVT and portal hypertension; short term in-hospital benefit was derived mainly from treatment of sepsis rather than anticoagulation.^[11]^

## 4. Conclusion

Abdominal variant of LS, although carries a high mortality rate with long term disease morbidity it can be adequately managed with intravenous antibiotics with or without anticoagulation following early diagnosis. Clinicians should, therefore, always be mindful of the condition especially in the presence of relevant risk factors with supportive intra-abdominal imaging findings. Furthermore, because of the rarity of the condition and lack of evidence-based treatment recommendations, management approaches for each patient should be individualized with involvement of multidisciplinary teams.

## Author contributions

**Conceptualization:** Aditya Keerthi Rayapureddy, Kapilkumar Manvar.

**Data curation:** Sagar Pandey, Aditya Keerthi Rayapureddy, Kapilkumar Manvar, Sushma Edara, Gouthami Boddu, Ajit Thakur, Vijay Jaswani.

**Formal analysis:** Sagar Pandey, Aditya Keerthi Rayapureddy, Kapilkumar Manvar, Sushma Edara, Gouthami Boddu, Ajit Thakur, Vijay Jaswani.

**Investigation:** Kapilkumar Manvar, Aditya Keerthi Rayapureddy, Sagar Pandey, Sushma Edara, Ajit Thakur, Vijay Jaswani.

**Methodology:** Sagar Pandey, Aditya Keerthi Rayapureddy, Kapilkumar Manvar, Vijay Jaswani.

**Resources:** Sagar Pandey, Aditya Keerthi Rayapureddy, Kapilkumar Manvar, Gouthami Boddu, Vijay Jaswani.

**Software:** Sagar Pandey, Ajit Thakur.

**Supervision:** Kapilkumar Manvar, Aditya Keerthi Rayapureddy, Sagar Pandey, Sushma Edara, Gouthami Boddu, Ajit Thakur, Vijay Jaswani.

**Writing – original draft:** Sagar Pandey, Aditya Keerthi Rayapureddy, Ajit Thakur.

**Writing – review & editing:** Sagar Pandey, Aditya Keerthi Rayapureddy, Kapilkumar Manvar, Sushma Edara, Gouthami Boddu, Ajit Thakur, Vijay Jaswani.
